# Supplementary material for: COVID-19 impact on global maritime mobility
Source: Sci Rep. 2021 Sep 10;11:18039. doi: 10.1038/s41598-021-97461-7 (PMC8433355; doi:10.1038/s41598-021-97461-7)
Supplement: Supplementary file 1 — Supplementary Information. [file 41598_2021_97461_MOESM1_ESM.pdf]

COVID-19 Impact on Global Maritime Mobility — Supplementary Information —

Leonardo M. Millefiori[1,+], Paolo Braca[1,+,*], Dimitris Zissis[2,3], Giannis Spiliopoulos[3], Stefano Marano[4], Peter K. Willett[5], and Sandro Carniel[1]

[1]NATO STO Centre for Maritime Research and Experimentation, Research Department, La Spezia, 19126, Italy
[2]University of the Aegean, Department of Product and Systems Design Engineering, Syros, 84100, Greece
[3]MarineTraffic, Athens, 115 25, Greece
[4]University of Salerno, Dipartimento di Ingegneria dell'Informazione ed Elettrica e Matematica Applicata (DIEM), Fisciano (SA), 84084, Italy
[5]University of Connecticut, Department of Electrical and Computer Engineering, Storrs, 06269, USA
[*]paolo.braca@cmre.nato.int
[+]these authors contributed equally to this work

ABSTRACT

In this document, we provide additional analysis on the impact of COVID-19 on maritime traffic.

Ship size analysis

For a closer look into each market, we report the daily navigated miles broken down by ship size. In Fig. S1, we compare the daily navigated miles for container ships according to their capacity. Specific segments show a stronger decline compared to others. Across all vessel sizes, with the exception of Ultra Large Container Vessels (ULCVs), from late February or early March, there is a strong decrease of the navigated miles. As before, we must note that, since specific segments and types of ships operate on specific trade routes and regions, there could be regional effects that, in the global indicators, are "averaged out"; in other words, local trends could be different than the global one. This is evident from the density map analysis, which show exactly how changes are spatially distributed. However, if considered globally, the ULCV market declined; ship operators cancelled their services, with a consequent decrease of both active ships and navigated miles. In Figures S2 and S3, a similar analysis is available for dry and wet bulk carriers. Dry bulk shipping refers to the movement of commodities carried in bulk: iron ore, coal, grain, steel products, lumber and other commodities classified as the minor bulks. For this category, only a small decrease of mileage across all vessel sizes is observed. In Fig. S3, we report the daily navigated miles for wet bulk shipping broken down by ship capacity. Wet bulk cargoes include petroleum products, crude oil, vegetable oils, chemicals and similar products. The slowing demand in goods' production and oil consumption had an effect on the mobility of all vessel sizes. The most significantly affected have been the larger tankers, specifically Panamax, Aframax and Suezmax sizes being affected the most; comprehensive information on ship classification by their size is available to the interested reader in the open literature.[1,2] Due to circumstances unrelated to COVID-19 (i.e., the breakdown of the OPEC alliance—which triggered a 30 % fall in oil prices in March 2020), the mobility reduction is evident only after April 2020. Finally, Fig. S4 reports the daily navigated miles of passenger vessels, the most affected segment by lockdown measures. The loss in terms of navigated miles in 2020 compared to 2019 is apparent, with larger vessel sizes affected by stronger losses than smaller ones. With respect to 2019, passenger ships larger than 60K GT, which includes large cruise ships, registered a sharp decrease of navigated miles of more than 80 % since March 2020, when several cruise lines, including Carnival cruises, which alone owns more than 100 cruise ships, suspended the operations. The effects are evident from Fig. S4, with a sharp drop of navigated miles apparent since March.

Daily average speed analysis

In Figures S5–S8, we report the daily average speed broken down by ship size and category. For the container, dry bulk and wet bulk categories, the average speed did not change significantly from January to June 2020. Instead, passenger ships exhibit a significant decrease in average speed, as reported in Fig. S8, a behavior that is coherent with the trend of Cumulative Navigated Miles (CNM) reported in Fig. S4. Figures S1–S3 show an increase of the average speed for several classes of container, dry bulk and wet bulk in May and June 2019, which is not observed in 2020. Again, this is coherent with the slowdown of the CNM in 2020 compared to 2019 for most of ship traffic, except dry bulk traffic, as reported in Figures S1–S3.

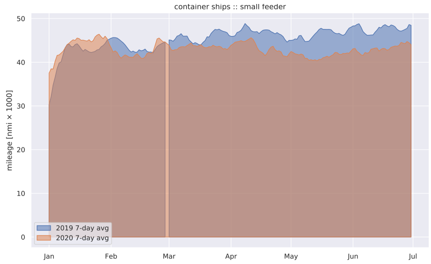

(a) Container ships: Small feeder

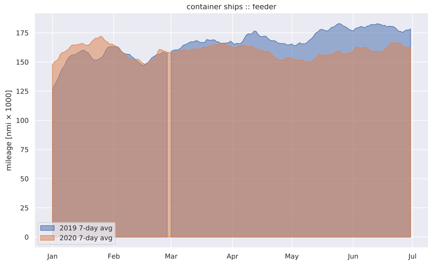

(b) Container ships: Feeder

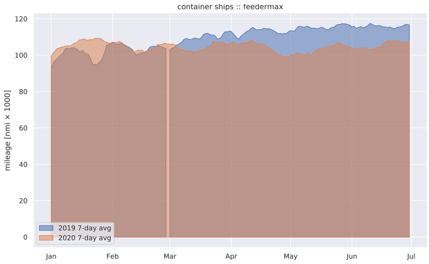

(c) Container ships: Feedermax

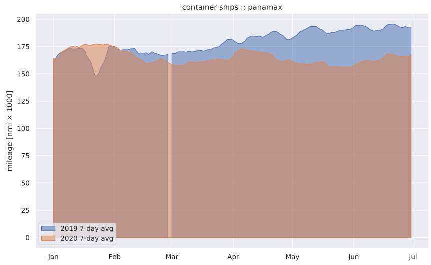

(d) Container ships: Panamax

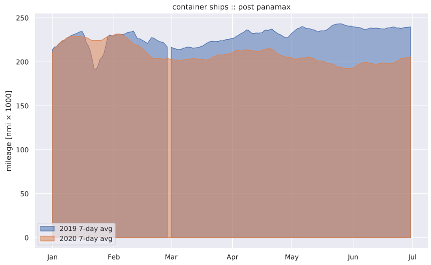

(e) Container ships: Post-Panamax

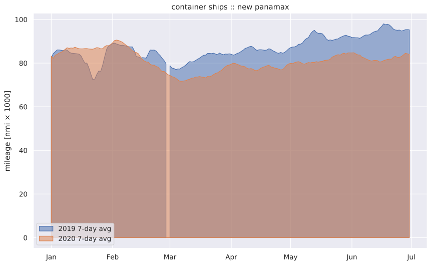

(f) Container ships: New Panamax

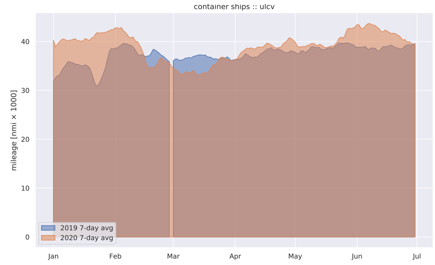

(g) Container ships: ULCV

Figure S1. Comparison of daily miles navigated by container ships in the first six months of 2020 (orange) versus 2019 (blue) for different ship size categories, ordered by increasing capacity measured in Twenty-foot Equivalent Units (TEUs); the two area charts are overlaid in transparency to highlight trend differences; the discontinuity in the blue data series corresponds to the leap day absence in 2019.

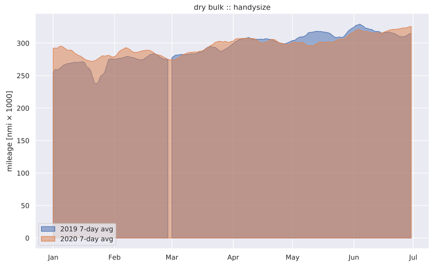

(a) Dry bulk: Handy size

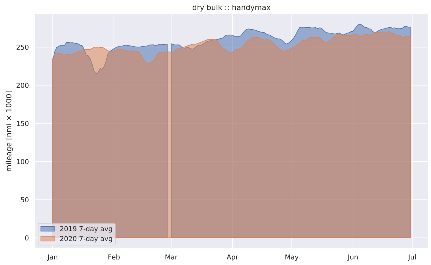

(b) Dry bulk: Handymax

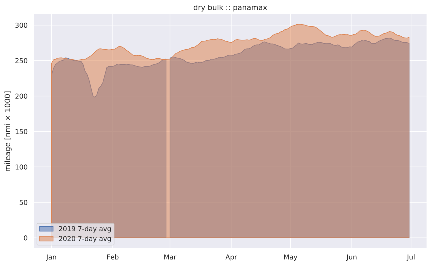

(c) Dry bulk: Panamax

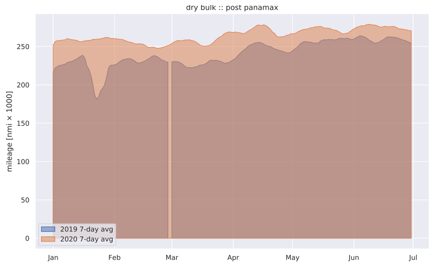

(d) Dry bulk: Post-Panamax

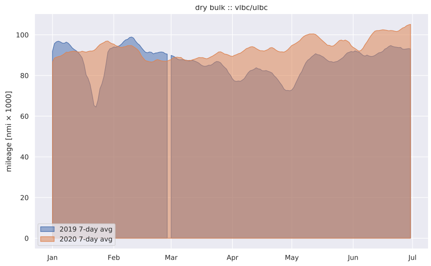

(e) Dry bulk: VLBC

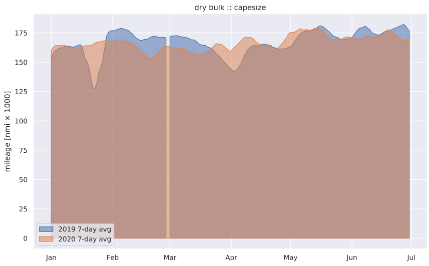

(f) Dry bulk: Capesize

Figure S2. Comparison of daily miles navigated by dry bulk ships in the first six months of 2020 (orange) versus 2019 (blue) for different ship size categories, ordered by increasing deadweight tonnage (DWT); the two area charts are overlaid in transparency to highlight trend differences; the discontinuity in the blue data series corresponds to the leap day absence in 2019.

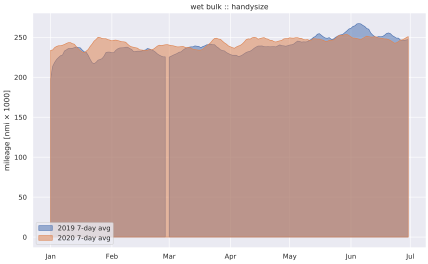

(a) Wet bulk: Handy size

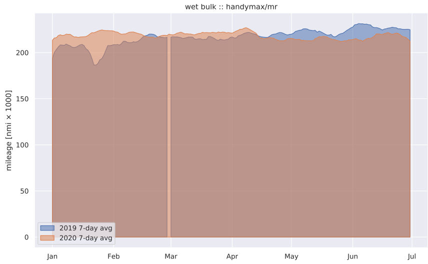

(b) Wet bulk: Handymax

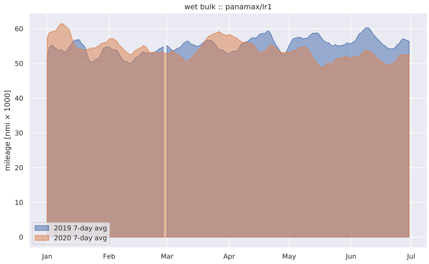

(c) Wet bulk: Panamax

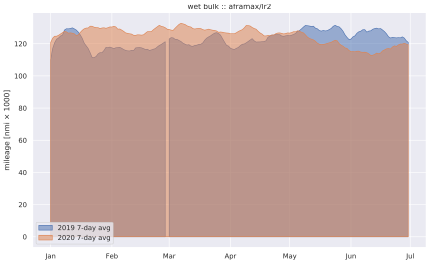

(d) Wet bulk: Aframax

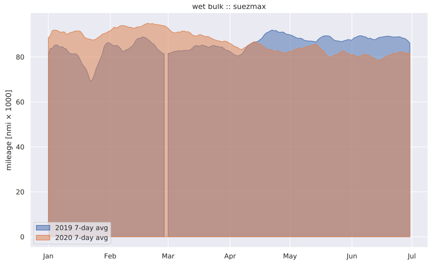

(e) Wet bulk: Suezmax

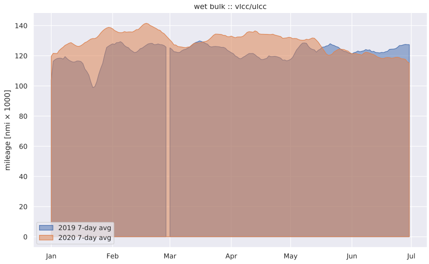

(f) Wet bulk: VLCC

Figure S3. Comparison of daily miles navigated by wet bulk ships in the first six months of 2020 (orange) versus 2019 (blue) for different ship size categories, ordered by increasing DWT; the two area charts are overlaid in transparency to highlight trend differences; the discontinuity in the blue data series corresponds to the leap day absence in 2019.

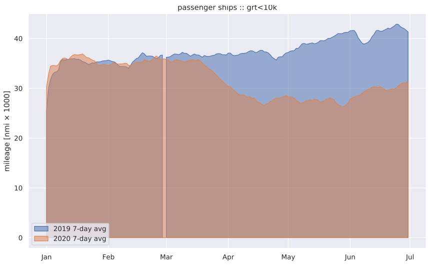

(a) Passenger ships: GT $\leq$ 10K

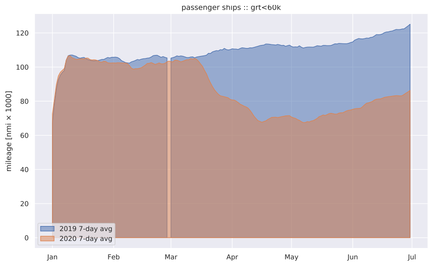

(b) Passenger ships: 10K $<$ GT $\leq$ 60K

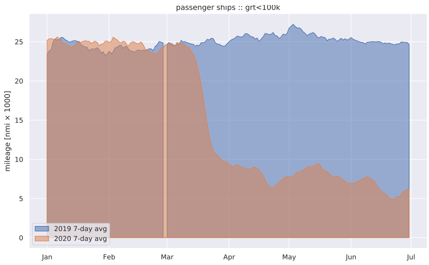

(c) Passenger ships: 60K $<$ GT $\leq$ 100K

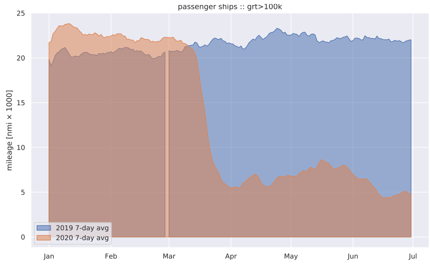

(d) Passenger ships: GT $>$ 100K

Figure S4. Comparison of daily miles navigated by passenger ships in the first six months of 2020 (orange) versus 2019 (blue) for different ship size categories, ordered by increasing gross tonnage (GT); the two area charts are overlaid in transparency to highlight trend differences; the discontinuity in the blue data series corresponds to the leap day absence in 2019.

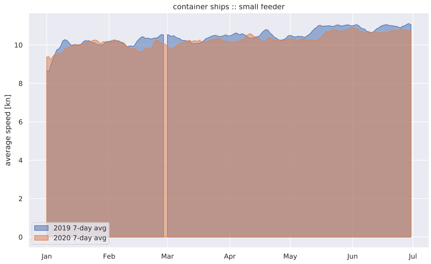

(a) Container ships: Small feeder

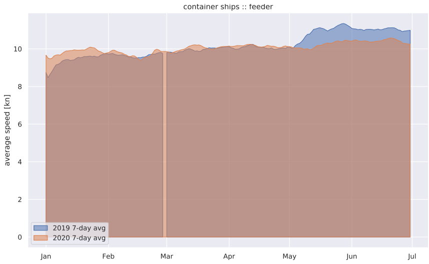

(b) Container ships: Feeder

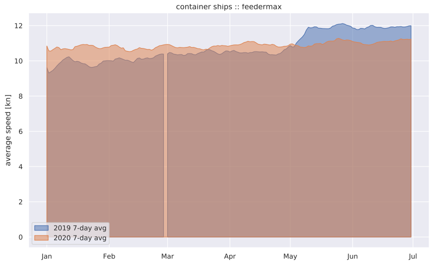

(c) Container ships: Feedermax

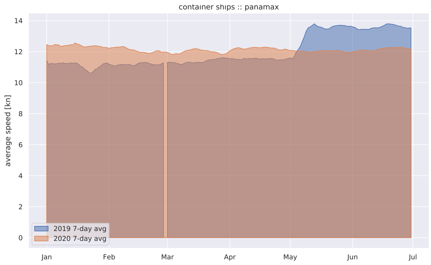

(d) Container ships: Panamax

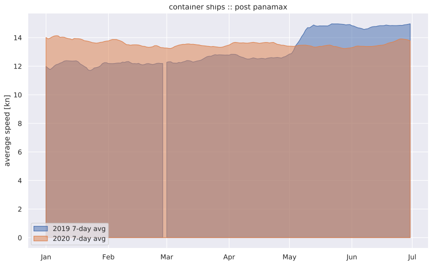

(e) Container ships: Post-Panamax

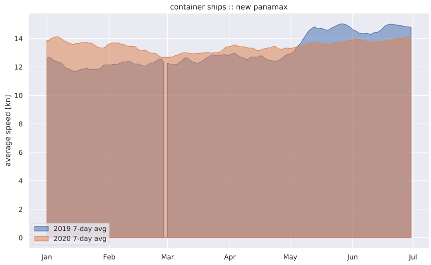

(f) Container ships: New Panamax

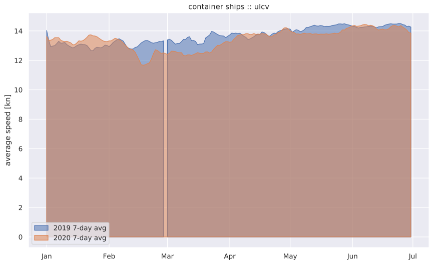

(g) Container ships: ULCV

Figure S5. Comparison of daily average speed of container ships in the first six months of 2020 (orange) versus 2019 (blue) for different ship size categories, ordered by increasing capacity measured in TEUs; the two area charts are overlaid in transparency to highlight trend differences; the discontinuity in the blue data series corresponds to the leap day absence in 2019.

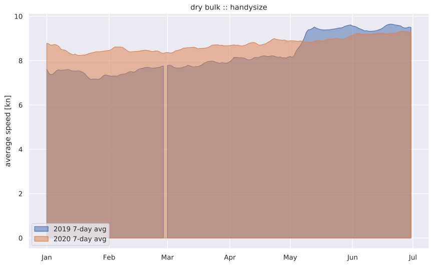

(a) Dry bulk: Handy size

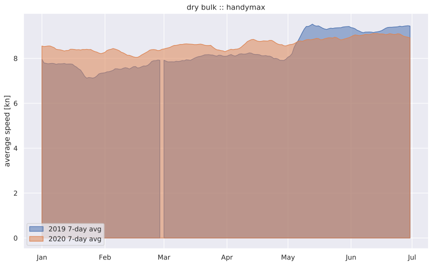

(b) Dry bulk: Handymax

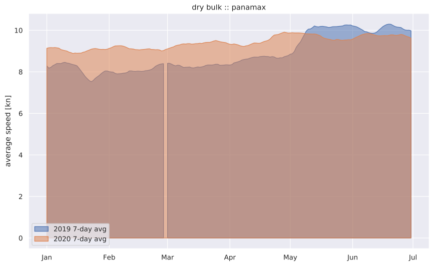

(c) Dry bulk: Panamax

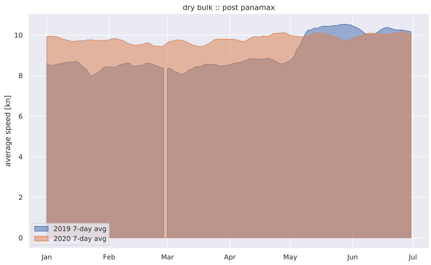

(d) Dry bulk: Post-Panamax

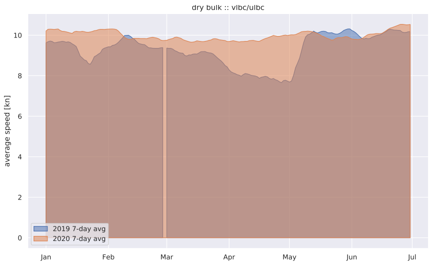

(e) Dry bulk: VLBC

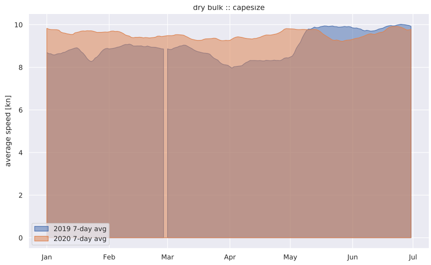

(f) Dry bulk: Capesize

Figure S6. Comparison of daily average speed of dry bulk ships in the first six months of 2020 (orange) versus 2019 (blue) for different ship size categories, ordered by increasing DWT; the two area charts are overlaid in transparency to highlight trend differences; the discontinuity in the blue data series corresponds to the leap day absence in 2019.

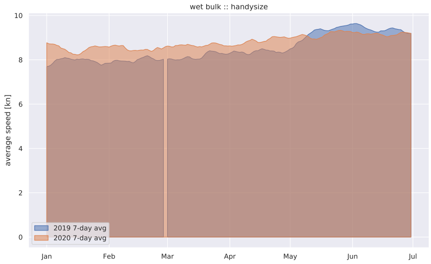

(a) Wet bulk: Handy size

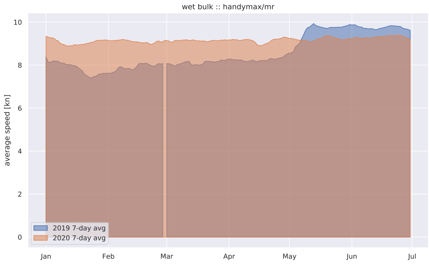

(b) Wet bulk: Handymax

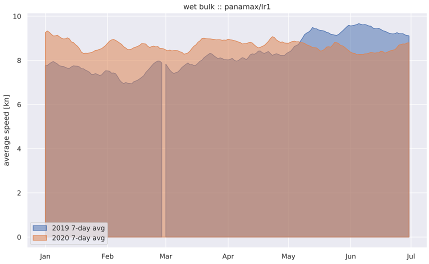

(c) Wet bulk: Panamax

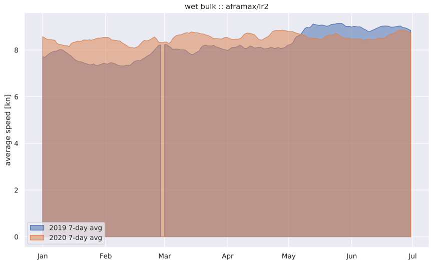

(d) Wet bulk: Aframax

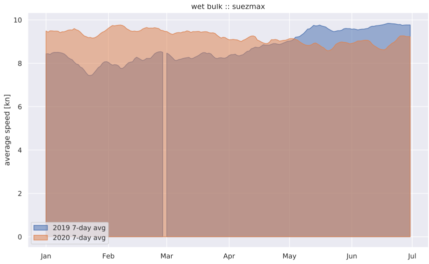

(e) Wet bulk: Suezmax

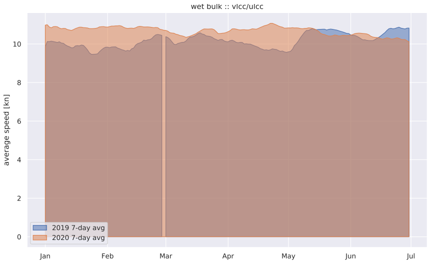

(f) Wet bulk: VLCC

Figure S7. Comparison of daily average speed of wet bulk ships in the first six months of 2020 (orange) versus 2019 (blue) for different ship size categories, ordered by increasing DWT; the two area charts are overlaid in transparency to highlight trend differences; the discontinuity in the blue data series corresponds to the leap day absence in 2019.

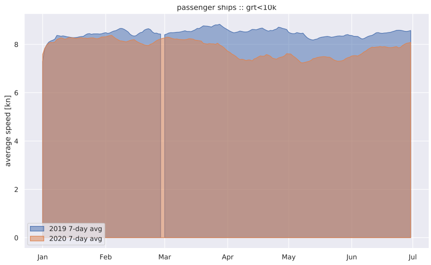

(a) Passenger ships: GT $\leq$ 10K

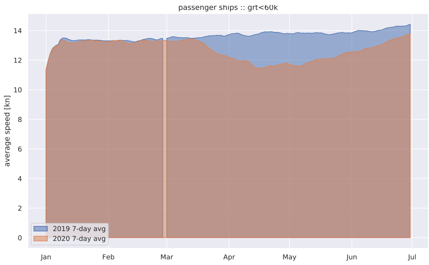

(b) Passenger ships: 10K $<$ GT $\leq$ 60K

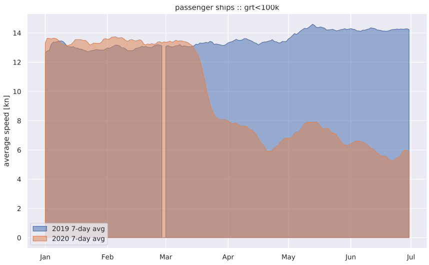

(c) Passenger ships: 60K $<$ GT $\leq$ 100K

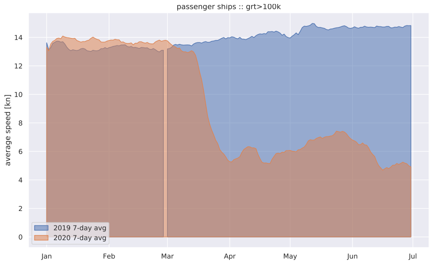

(d) Passenger ships: GT $>$ 100K

Figure S8. Comparison of daily average speed of passenger ships in the first six months of 2020 (orange) versus 2019 (blue) for different ship size categories, ordered by increasing gross tonnage (GT); the two area charts are overlaid in transparency to highlight trend differences; the discontinuity in the blue data series corresponds to the leap day absence in 2019.

References

1. MAN Diesel & Turbo. Propulsion trends in container vessels. https://web.archive.org/web/20120507192232/http://www.mandieselturbo.eu/files/news/filesof4672/5510-0040-01ppr_low.pdf (2009).
2. MAN Diesel & Turbo. Propulsion trends in bulk carriers. https://web.archive.org/web/20200902103322/https://marine.man-es.com/docs/librariesprovider6/test/propulsion-trends-in-bulk-carriers.pdf (2019).
